# Sirtuins of parasitic protozoa: In search of function(s)

**DOI:** 10.1016/j.molbiopara.2012.08.003

**Published:** 2012-10

**Authors:** Agnieszka A. Religa, Andrew P. Waters

**Affiliations:** Wellcome Trust Centre for Molecular Parasitology, Institute of Infection, Immunity and Inflammation, University of Glasgow, Glasgow G12 8TA, UK

**Keywords:** *sir2*, Parasite, *Plasmodium*, *Leishmania*, Gene regulation, Antigenic variation

## Abstract

The SIR2 family of NAD^+^-dependent protein deacetylases, collectively called sirtuins, has been of central interest due to their proposed roles in life-span regulation and ageing. Sirtuins are one group of environment sensors of a cell interpreting external information and orchestrating internal responses at the sub-cellular level, through participation in gene regulation mechanisms. Remarkably conserved across all kingdoms of life SIR2 proteins in several protozoan parasites appear to have both conserved and intriguing unique functions. This review summarises our current knowledge of the members of the sirtuin families in Apicomplexa, including *Plasmodium*, and other protozoan parasites such as *Trypanosoma* and *Leishmania*. The wide diversity of processes regulated by SIR2 proteins makes them targets worthy of exploitation in anti-parasitic therapies.

## Graphical abstract

**Graphical abstract:**

**Figure:**
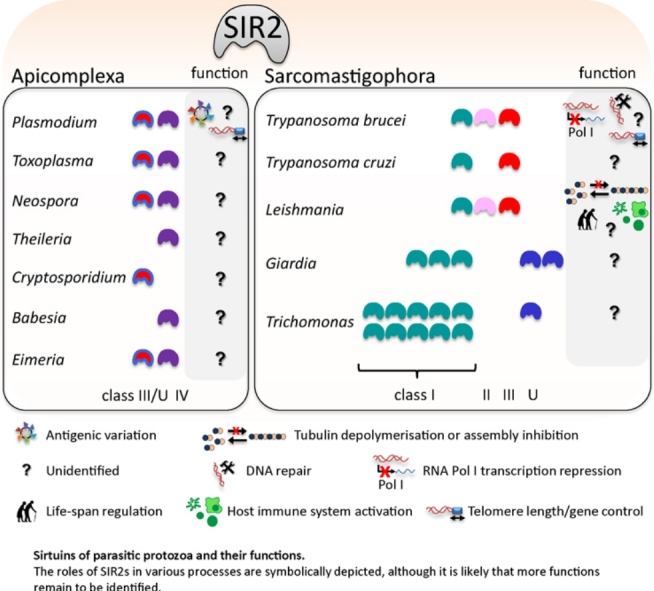

**Highlights:**

► General concise introduction into the world of SIR2s. ► Overview of the SIR2 family members’ representation in parasitic protozoa, with phylogenetic classification. ► Summary of findings on SIR2 proteins structure, function and localisation in parasitic protozoa. ► Mention of potential therapies stemming from SIR2 studies in parasitic protozoa.

## 1 The sirtuin family – introduction

Recently a wealth of data has emerged that implicates protein acetylation as a major (and frequently overlooked) cellular mechanism to control protein activity through post-translational modification. Protein acetylation is well characterised in the case of chromatin (*e.g.* histones), and acetylated proteins are highly abundant in the nucleus with targets including chromatin modifying enzyme complexes affecting all major nuclear processes. Furthermore, protein acetylation appears to play a central role in many cytoplasmic events, such as protein folding, signal transduction, cell cycle control and cytoskeletal regulation. The acetylation status of a target protein is determined by the action of protein acetylases and deacetylases – a large family of diverse proteins that includes the sirtuin proteins which are the focus of this review.

The silent-information regulator 2 (SIR2)-like family of nicotinamide nucleotide dinucleotide (NAD^+^)-dependent protein deacetylases, commonly called sirtuins [1,2], are highly conserved from archaea to higher eukaryotes, and are arguably most renowned for their role in longevity. Sirtuins have a wide scope of cellular actions and have been in the spotlight of ageing, life-span and metabolism studies and are linked to a number of diseases, including Alzheimer's disease, obesity, type II diabetes, neurodegenerative disorders and cancer (recent reviews see *e.g.* [3–7]). At the cellular level sirtuins collectively participate in many cell activities including but not restricted to gene silencing, cell cycle progression, chromosome segregation, microtubule organisation, protein aggregates transport, genome stability, DNA repair, apoptosis, autophagy (for reviews see for instance [8,9]).

Since sirtuins are central to proper cell functioning and proliferative life span, their role in pathogenic organisms such as those contained within the Apicomplexa is intriguing. This review will summarise the recent advances in the study of sirtuins in a number of parasitic protozoa of medical and veterinary importance, comparing these findings with those from other organisms where they have been described in some detail. Sirtuins in parasitic protozoa have both the canonical and atypical activities that contribute to both conserved and apparently unique functions. The biological implications and available sirtuin-targeting drugs, which could be used in treatments of parasitic disease are also discussed.

## 2 The phylogenetic distribution of sirtuins

**Fig. 1:**
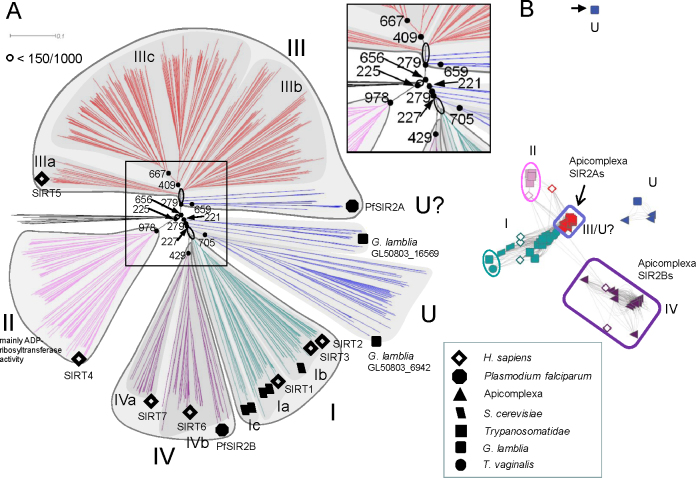
Sirtuin phylogeny with focus on parasitic protozoa. (A) A phylogenetic tree of 778 annotated sirtuins from different organisms (sources NCBI, EuPathDB, GeneDB) produced in ClustalX2 using Neighbour-Joining (NJ) method, with a bootstrap value of 1000. The phylogenetic tree branches were coloured according to the previous classification into 5 main clades: class I–IV (teal, pink, red and purple, respectively) and class U (blue) [10]. Note that several blue branches including *Plasmodium* SIR2As are assigned to group III (red) (as previously described in *e.g.*[10,12,13]) though with weak support. Therefore these sirtuins could also be grouped with class U sirtuins. Human sirtuin family members are marked with full diamonds. Incomplete protein sequences were removed from the analysis to facilitate the phylogenetic tree construction (for a list of sirtuins see Suppl. Table 1). The inset shows an enlargement clarification of the bootstrap values supporting particular nodes. (B) A Sequence Similarity Network (SSN) for the parasitic protozoa extracted from a global SSN built on BLAST pairwise alignments of sirtuin sequences from (A), with each node representing a single sirtuin and BLAST e-values as edges (cut-off threshold e^15^). The resulting SSN network intuitively represents the sirtuins class divisions (same colours as in B). Sirtuins of the parasitic protozoa contribute to every class of sirtuins. Apicomplexa sirtuins (triangles) belong mainly to class III/U and IV sirtuins (see text for details). *H. sapiens* (diamonds) and *S. cerevisiae* (parallelograms) SIRs are shown, and cluster accordingly to the previous classification [10]. (For interpretation of the references to color in this figure legend, the reader is referred to the web version of the article.)

Initial phylogenetic analysis classified 60 sirtuins into five main types based on the different sirtuin domains of the 7 human sirtuins (hSIRT1–7) [10] with type IV subdivided into IVa and IVb, and type I into a, b and c. A very recent phylogenetic analysis of 240 sirtuins confirmed these classifications and proposed further subdivisions of Class III (a, b and c) and split the undifferentiated (U) category into U1–U4 branches [11]. Our more extensive phylogenetic tree is based on Neighbour-Joining (NJ) method, for more than 700 sirtuin sequences from 529 organisms including the parasitic protozoa (Fig. 1A and see Suppl. Table 1 for a list of sequences originating from parasite genomes). The different protozoan parasite genomes are predicted to contain one or multiple sirtuin genes (see below). The global tree greatly resembles the earlier analyses with class I–IV (colours: teal, pink, red and purple, respectively), and class U (blue) (subclasses of the U class are not yet supported). Sequence similarity networks (SSN; Fig. 1B) incorporate not only aligned sequences but may be combined with experimental data such as substrate specificity and subcellular location to derive their graphical output. This provides a more practical visualisation of functional relationships between members of a protein family and emphasises the diverged nature of the unclassified members. Fig. 1B represents putative sirtuins of parasitic protozoa and their relative distances obtained from blasting the parasitic protozoa sequences against all the sequences used in defining the canonical sirtuin domain (PFAM ID: PF02146; http://pfam.sanger.ac.uk/). Parasite sirtuins are distributed in all of the phylogenetically defined sirtuin classes. Class I sirtuins (coloured green) are found only in Sarcomastigophora (includes the Trypansomatids) and not in Apicomplexa (see Table 1 for details). Although a *Trichomonas vaginalis* sirtuin (TVAG_146810) and a *Giardia lamblia* sirtuin (GL50803_11676) are relatively distant from the remaining parasitic protozoan sirtuins in that class. Class II (coloured in pink) contains one of the trypansomatid sirtuins from the three *Leishmania* species (LmjF23.1210, LbrM23_V2.1310, LinJ23_V3.1450) and *Trypanosoma brucei* (Tb927.8.3140; circled in pink in Fig. 1B). Most apicomplexans possess 2 sirtuins: SIR2A and SIR2B (triangles). SIR2A sirtuin domains can be assigned to class III (coloured red) with moderate support (as previously described in *e.g.* [10,12,13]), or class U (hence coloured red circled in blue). Apicomplexa SIR2B sirtuin domains all cluster very well with class IV sirtuins (purple box) together with human SIRT6 and SIRT7 (human SIRT1–7; Fig. 1B diamonds). Perhaps unsurprisingly, a sirtuin of the ancient protist *G. lamblia* (GL50803_6942) appears to be the most distant example of protozoan sirtuins analysed and groups with putative sirtuins of archaebacteria possibly reflecting the proposed ancient origin of *Giardia*. Another *G. lamblia* SIR2 (GL50803_16569) forms an outlying cluster (circled in black), with the single class U SIR2 predicted in the *Cryptosporidium* spp. genomes (triangles). Human sirtuins typify classes I–IV. Yeast SIR2s as well as hSIRT1–4 all cluster into class I (in agreement with the original classification [10]).

Table 1 lists parasitic protozoa shown in Fig. 1B, and number and classification of sirtuins found in their genomes. As previously mentioned Apicomplexa possess only 1–2 SIRs (based on available sequence data; see data source column, Table 1), whereas the remaining characterised parasitic protozoan lineages apparently have more. For instance based on the current genome assembly (scaffold genome 01/2007 [14]; search performed on TrichDB v1.3 on 08/2011) of *T. vaginalis* as many as 11 distinct sirtuins are found in the genome (TrichDB, blast analysis using *S. cerevisiae* SIR2/YDL042C as query) although this prediction may yet reflect the incomplete nature of the genome sequence and its annotation. In summary sirtuin domains of parasitic protozoa are widely dispersed across the sirtuin classes. Nevertheless the sirtuin domain is structurally highly conserved as shown below indicating general functional conservation, *i.e.* involvement in NAD^+^-dependent protein acetylation and/or ADP ribosylation.

## 3 Structure

**Fig. 2:**
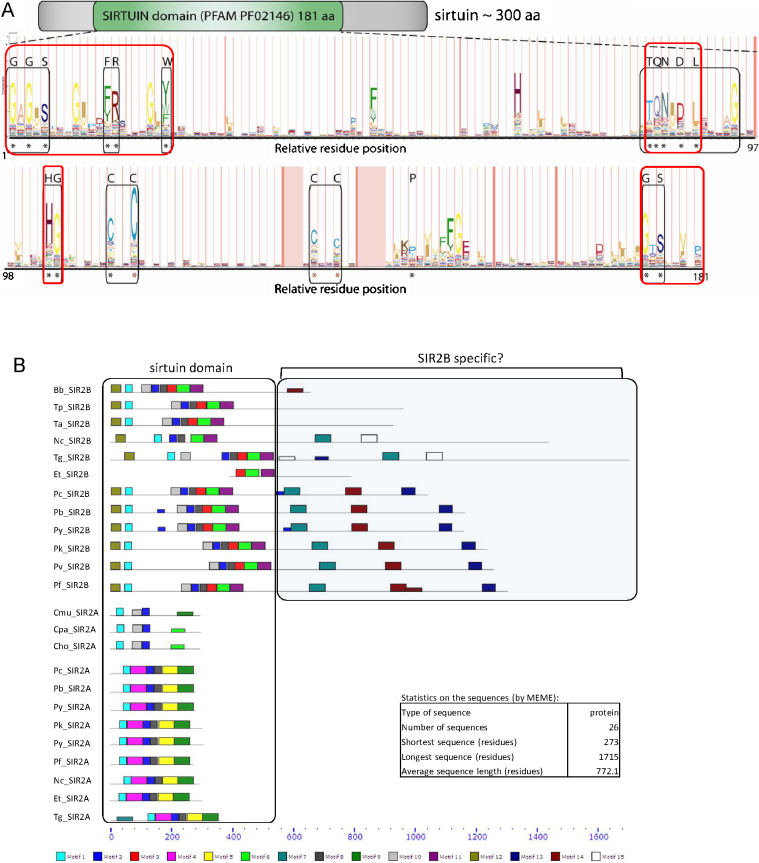
Structural organisation of the sirtuin domains of parasitic protozoa. (A) The average sirtuin of approximately 300 amino acids contains the canonical sirtuin domain (PFAM model PF02146) of 181 aa with several conserved regions (lower panel). Asterisks indicate residues conserved among parasitic protozoa according to ClustalX alignments (black = perfectly conserved, red = highly conserved). (B) Protein alignment of all Apicomplexa sirtuins with indicated subdomains characterised *in silico* by MEME sequence analysis tool (http://meme.sdsc.edu). The search was limited to 15 motifs/subdomains of between 6 and 50 aa. Sirtuin domain alignment (ClustalX2) with highlighted subdomains identified by MEME shows several highly conserved regions, with perfectly conserved residues within motif 1 (G[AS]GXS, FR), motif 2 (TQN[IV]D[SGN]L) and motif 8 (HG,CXXC). Motifs 7, 13 and 14 are present in most Apicomplexa SIR2B-like sirtuins, including all *Plasmodium* SIR2Bs, *B. bovis* SIR2B (Bb_SIR2B), *N. caninum* Nc_SIR2B, *T. gondii* Tg_SIR2B. Interestingly *Cryptosporidium* spp. sirtuins posesess some similarity in motif composition to Apicomplexa SIR2Bs. Note that *Eimeria tenella* Et_SIR2B sequence appears incomplete. Bb = *Babesia bovis*, Et = *Eimeria tenella*, *Cryptosporidium*: Cmu = *C. muris*, Cpa = *C. parvum*, Cho = *C. hominis*, Nc = *Neospora caninum*, *Plasmodium*: Pc = *P. chabaudii*, Pb = *P. berghei*, Py = *P. yoelii*, Pk = *P. knowlesi*, Pv = *P. vivax*, Pf = *P. falciparum*, Ta = *Theileria annulata*, Tp = *Theileria parva*, Tg = *Toxoplasma gondii*. (C) *Top*: 3D structure alignment of hSIRT5 and PfSIR2A (PDB IDs: 2B4Y gold and 3JWP blue respectively) using PyMOL (DeLano Scientific, www.pymol.org), structural alignment root mean squared (RMS) value = 2.0. PfSIR2A: The structure is shown with adenosine monophosphate (AMP) present in the NAD^+^-binding pocket in the Rossman fold (coloured red). hSIRT5: The structure is shown with adenosine-5-diphosphoribose (ADP) present in the NAD^+^-binding pocket in the Rossman fold (coloured green). The co-ordinated zinc ion is shown as grey (PfSIR2A) and black (hSIRT5) spheres. *Bottom*: 3D structure alignment of a class U sirtuin from *Thermotoga maritima* and PfSIR2A (PDB IDs: 3JR3 purple and 3JWP teal respectively). RMS value = 1.7. PfSIR2A: The structure is shown with adenosine monophosphate (AMP) present in the NAD^+^-binding pocket in the Rossman fold (coloured red). TmSIR2: The structure does not contain molecules in the NAD^+^-binding pocket. The co-ordinated zinc ion is shown as grey (PfSIR2A) and black (TmSIR2) spheres. (For interpretation of the references to color in this figure legend, the reader is referred to the web version of the article.)

Sirtuin family members share a catalytic domain that allows the majority of sirtuins to function as NAD^+^-dependent protein deacetylases. However the same domain generally acts as ADP-ribosyltransferase in some cases exclusively so and this specialised type of sirtuin appears currently restricted to class II sirtuins (which includes bacterial and human SIRT4, see Fig. 1A). A typical sirtuin (shown in Fig. 2A, see also Fig. 2C) largely consists of a sirtuin catalytic domain, which in turn comprises both a NAD-binding and acetyl-lysine-binding domain (red boxes in Fig. 2A), as well as a variable zinc ion-binding domain implicated in substrate specificity of different sirtuin proteins. The PFAM database defines the canonical sirtuin domain (PFAM PF02146) which consists of several highly conserved subdomains stretching over 181 amino acids. Sirtuin domains of parasitic protozoa (database IDs in Table 1) conform to the canonical sirtuin domain, including several of the highly conserved regions (Fig. 2A black boxes). A number of residues are perfectly conserved within Apicomplexa (asterisked) which is further strengthened by MEME motif analysis (http://meme.sdsc.edu; Fig. 2B) of all Apicomplexa sirtuins, which demonstrated localised high conservation within the sirtuin domain. The Class IV type SIR2Bs apicomplexan SIR2Bs (*Plasmodium*, *Toxoplasma*, *Neospora*, *Theileria*, *Babesia* and *Eimeria*) all contain several extra apparently SIR2B-specific motifs in addition to the canonical sirtuin domain, with the exception of *Eimeria tenella* SIR2B (Eth_SIR2B) which appears incomplete in sequence. The functional significance of SIR2B-specific motifs awaits characterisation. The sirtuin domain flanking regions typically determine the functional context such as subcellular localisation and protein–protein interactions modulating substrate range [15]. For instance, mammalian SIRT1 exhibits different modes of nucleo-cytoplasmic shuttling depending on tissue or cell type. Two N-terminal nuclear localisation signals (NLS) and two nuclear export signals (NES) functionally identified in mouse SIRT1 [16] and found in SIRT1 from other organisms (nematodes, flies, other mammals) dictate various SIRT1 localisations and thus actions, such as protection from apoptosis (when nuclear) or induction of cell motility (when cytoplasmic). Similar features are starting to be demonstrated in parasitic sirtuins (see below).

Little is known of the tertiary structure of parasitic sirtuins. However a recently solved crystal structure of PfSIR2A at a resolution of 2.65 Å (PDB ID: 3JWP; Wernimont et al., unpublished) demonstrated that the structure is similar to that of class III human SIRT5 (PDB ID 2B4Y; Antoschenko et al., unpublished) despite only sharing 31% aa identity (Fig. 2C top panel), or to *Thermotoga maritima* class U SIR2 (TmSIR2, PDB ID 3JR3; Howse and Wolberger, unpublished) with 33% aa identity (Fig. 2C bottom panel). The PfSIR2A protein consists of the catalytic sirtuin domain with the Rossman fold, typical in proteins with binding affinities for nucleotides including NAD (multiple Rossman folds) or flavin mononucleotide (FMN; single fold), and a variable domain that nevertheless contains the conserved Zn^2+^-coordinating cysteine residues (Fig. 2C, Zn^2+^ ions represented by a grey and black sphere for PfSIR2A and hSIRT5/TmSIR2, respectively). The Zn^2+^ metal ion coordination in sirtuins is mediated by two to four cysteines [17]. Recombinant PfSIR2A binds the zinc ion with high affinity at 1:1 stoichiometry indicating that PfSIR2A has a single Zn^2+^-binding site. Zinc binding is essential for the recombinant PfSIR2A structural integrity and deacetylase activity *in vitro* [17] which is typical of this class of deacetylase.

## 4 SIR2 functions and localisation

Histone deacetylation is performed by the majority of SIR2 proteins and is to date the most studied enzymatic activity of the sirtuin family. Histone deacetylation (of N-terminal lysine residues) is strongly linked to transcription repression and therefore has earned all histone deacetylases (HDACs) a description of transcriptional repressors. HDACs are conventionally divided into four classes, and the sirtuin family falls into class III deacetylases. The significant difference between sirtuins and the other three classes of HDACs is the dependency of sirtuins on the cofactor NAD^+^ (and thus potential inhibitor profile) and their unique insensitivity to the action of the general HDAC inhibitor trichostatin A (TSA). Sirtuins possess a second activity, first demonstrated in bacterial sirtuins, namely ADP-ribosyl transferase (ADPRT) activity involved in cobalamine biosynthesis [18], a finding subsequently broadened to eukaryotic sirtuins [19,20]. The latter study also showed that the ADPRT activity of SIR2 is essential for gene silencing in yeast. Subsequently, the N-terminal domains of histones H3 and H4 were found to be ADP-ribosylated by yeast and mouse SIR2 proteins, though only when already acetylated [21]. The requirement for an already acetylated histone substrate prior to ADP-ribosylation is not absolute; one of the *Trypanosoma brucei* sirtuins TbSIR2RP1 was shown to perform ADP-ribosylation reaction regardless of the acetylation status of the histone substrate. However in the absence of acetylation arginine (and not lysine) residues were ADP-ribosylated [22]. Furthermore, cysteine-specific mitochondrial ADP-ribosylation has been reported for hSIRT4, and this regulates glutamate dehydrogenase (GDH) activity [23,24]. It is however important to note that the ADP-ribosylation reaction usually occurs orders of magnitude more slowly than deacetylation [25, 26, and references therein] *e.g.* yeast SIR2 deacetylation is 1000 fold faster than ADP-ribosylation [27]. However, at least two of the mammalian sirtuins, namely SIRT6 and SIRT4 are predominantly ADPRTs having negligible deacetylase activity [23,28] although this may reflect use of unrecognised substrates (see below). Currently, the role of mono ADP-ribosylation of proteins in cellular processes and the interplay between ADP-ribosylation and other modifications remains to be fully uncovered. Two recently discovered activities of sirtuins include further modifications of lysine residues n proteins, namely demalonylation and desuccinylation (Kmal and Ksucc, respectively), as shown both *in vitro* and *in vivo* using mice [29–31]. The only enzyme to perform the two reactions is the mitochondrion-localised SIRT5 [29,30]. None of the remaining mammalian sirtuins (SIRT1–SIRT4, SIRT6, SIRT7) showed the demalonylase and desuccinylase activity [29,30]. The preference of SIRT5 for malonyllysine and succinyllysine is apparently due to an arginine and a tyrosine residue (Arg^105^, Tyr^102^) in the acyl pocket of the enzyme [29], and while SIRT5 also exhibits deacetylase activity the catalytic efficiency for demalonylation and desuccinylation is 29 → 1000 fold higher than for deacetylation [29]. Both the demalonylation and desuccinylation activities are NAD^+^-dependent and sensitive to nicotinamide inhibition but not TSA or sirtinol (see Section 6.1) [30]. The previously found deacetylation target of SIRT5, carbamoyl phosphate synthase 1 (CPS1) was also shown to be desuccinylated by SIRT5 *in vivo* [29,32]. These novel functions identified for SIRT5 open a new area of research on sirtuins plausibly acting as deacylases rather than just deacetylases in other organisms and perhaps restricted to a particular organelle. The capacity of sirtuins to perform several different post-translational modifications extends still further the complexity of cellular processes regulation and the possible role(s) of sirtuins therein. It is a possibility that protozoan parasite sirtuins might exhibit the demalonylation and desuccinylation functions, and this remains to be explored.

**Fig. 3:**
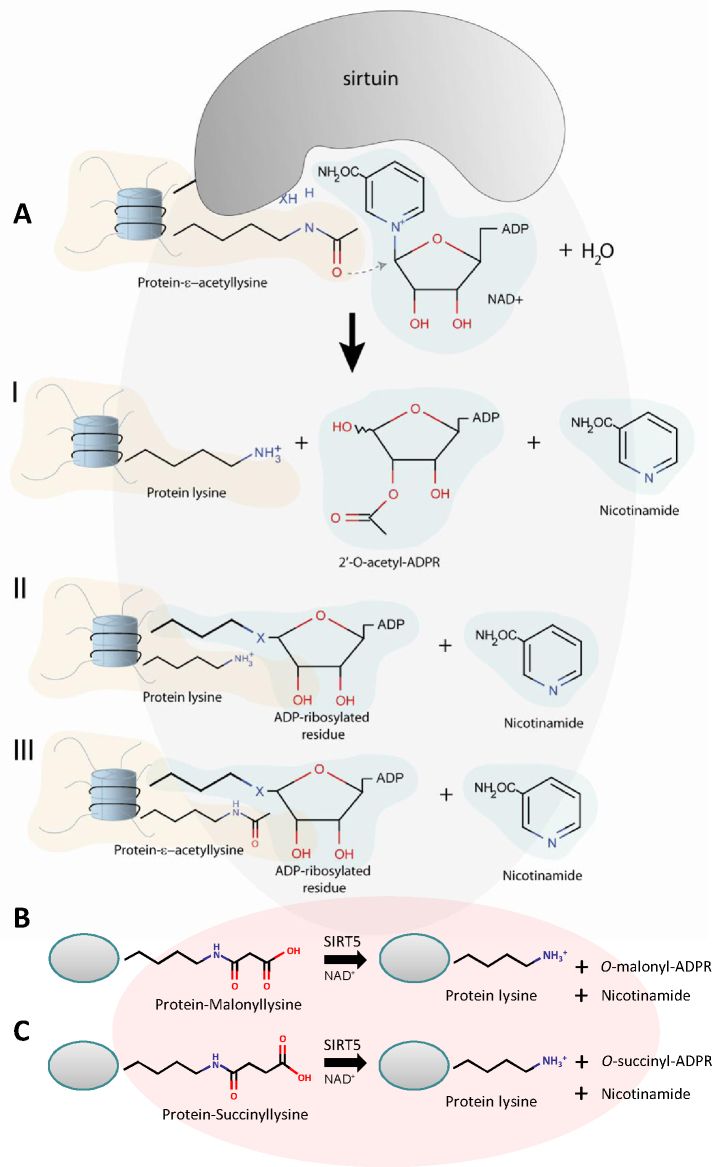
A simplified mechanism of sirtuin deacetylation reaction. (A) An acetylated lysine (K) residue on a histone protein (red background) is a sirtuin substrate. Nicotinamide adenine dinucleotide (NAD^+^; blue background) and H_2_O are necessary for the reaction. Several reactions are possible within a sirtuin active site, yielding: deacetylated K residue, nicotinamide and 2′-*O*-acetyl-ADP-ribose, a potential second messenger (products I), deacetylated K residue, an ADP-ribosylated residue and nicotinamide (products II), or acetylated K residue, an ADP-ribosylated residue and nicotinamide (products III). Reactions B and C are demalonylation and desuccinylation of K residues performed by SIRT5 and not for other mammalian sirtuins. Both B and C are NAD^+^-dependent and analogously to A produce nicotinamide and *O*-malonyl-ADP-ribose and *O*-succinyl-ADP-ribose, respectively. (For interpretation of the references to color in this figure legend, the reader is referred to the web version of the article.)

The mechanism by which sirtuins perform the deacetylation reaction is conserved and well-established [33]. However, the exact sequence of chemical events is still under debate, with some reports supporting the presence of an intermediate compound (S_N_1 reaction type) and others pointing to direct conversion of the substrates into reaction products (S_N_2 type). Nevertheless, deacetylation of an acetyllysine residue and NAD^+^ hydrolysis occurs in the same pocket of the enzyme, yielding a lysine residue, nicotinamide and *O*-acetyl-ADP-ribose (*O*AADPR) as the final three products (Fig. 3A, products I) only two products are generated by the ADP-ribosyltransferase (ADPRT) activity (see ADP-ribosylation reaction products Fig. 3A, II and III). The recently discovered and to date solely SIRT5-specific demalonylation and desuccinylation reactions occurring at lysine residues of a target protein are schematically depicted in Fig. 3 reactions B and C. Analogous to the deacetylation mechanism demalonylation and desuccinylation reactions produce nicotinamide, *O*-malonyl-ADP-ribose (*O*MaADPR) or *O*-succinyl-ADPR (*O*SuADPR) respectively [29].

**Fig. 4:**
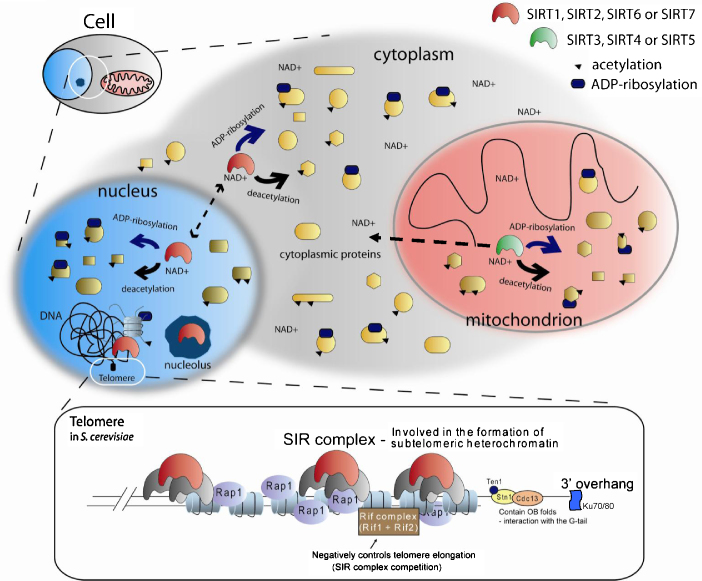
General function and localisation of sirtuins. Sirtuins function as NAD^+^-dependent deacetylases and/or ADP-ribosylases affecting a variety of cellular proteins and processes. Commonly in cells of higher mammals the seven sirtuins (SIRT1–SIRT7) localise to the nucleus and nucleolus (light blue and dark blue structures), cytoplasm (grey) and mitochondrion (red). A number of proteins (various yellow shapes), which can be acetylated (black triangle) are sirtuin targets of deacetylation or ADP-ribosylation (indicated by dark blue elipse). Depending on the sirtuin type, tissue/cell type and current cellular state different sirtuin family members localise to various cellular compartments, with possible shuttling *e.g.* between the nucleus and cytoplasm as indicated by the dashed double-headed arrow. SIRT3 as the only mitochondrial sirtuins has recently been reported localised in the cytoplasm and nucleus under stress conditions (dashed double-headed arrow) [166]. The nuclear sirtuins are involved in heterochromatin formation at several genomic locations, including telomeres (representation in the black box). In *S. cerevisiae* SIR2, SIR3 and SIR4 form a silencing complex at subtelomeric region. Heterochromatin formation is initiated by other proteins such as dsDNA-binding protein Rap1 and Ku70/Ku80 anchoring complex. Rif1–Rif2 compete for binding with the SIR complex. One of many other proteins at telomeres is Cdc13, here depicted interacting with Stn1–Ten1 proteins. This complex, binding to the G-rich 3′ overhang through OB domains, serves a protective function but also negatively regulates telomere elongation. (For interpretation of the references to color in this figure legend, the reader is referred to the web version of the article.)

In common with all HDACs, sirtuins were shown to be active beyond the nucleolus/nucleus and deacetylate not only histones but also other (cytoplasmic/mitochondrial) proteins (see Fig. 4 for a schematic representation), thereby regulating substrate activity and localisation. Therefore sirtuins exert their functions through a vast number of interacting proteins, which in turn mediate and control a wide scope of cellular processes occurring as a result of both cell-external and cell-internal signals. The interaction partners documented to date include FOXO proteins, p53, tubulin, pachytene checkpoint 2 (PCH2), Werner syndrome protein (WRN), peroxisome proliferator-activated receptor gamma coactivator-1 α (PGC-1α), acetyl-CoA synthetase 1, NF-κB, MEF2, *Xeroderma pigmentosum* group A (XPA), retinoic acid receptor β (RARβ), hypoxia-inducible factor 1 α (HIF-1α), endothelial nitric oxide synthase (eNOS), Alba proteins, Dishevelled (Dvl) proteins, HSP90 to name a few [34–42].

A number of studies have provided a link between transcription levels and the availability of nutrients (for a review see [43]). Sirtuins were proposed to act through “sensing” the intracellular levels of NAD^+^ which is greatly affected by calorie uptake (see reviews [44,45]). Therefore, since SIR2-like proteins are NAD^+^-dependent histone deacetylases and can shuttle between various cellular compartments, sirtuins are good candidates to effect and relay the status of external environmental nutrients to the different compartments of the cell interior. Increased SIR2 activity resulting from high NAD^+^ levels would be predicted to not only lead to decreased gene transcription at targeted loci (*e.g.* telomere-proximal “aging” genes suppression), but also protect the genome from free electrons or reactive oxygen species (ROSs) as well as protect the genome, including telomeres from undesirable recombination; all of which leads to enhanced genome and cellular stability. For instance, yeast SIR2 (ySIR2) was shown to increase genome stability through suppression of double-stranded breaks (DSBs) [46]. Both mammalian SIRT1 and SIRT6 are recruited to the DSBs facilitating repair through homologous recombination [47,48].

The functions of sirtuins in environment-affected cellular ageing seem to extend beyond the well-studied transcriptional and deacetylation effects in the nucleus. In *S. pombe* and *S. cerevisiae* SIR2 was shown to play a role in mitotic transport of sub-optimally folded protein aggregates from budding daughter to mother cells, which undergo ageing-like changes – a process known as spatial quality control – which is essential to keep the growing daughter cells pristine [49–52]. A multiprotein complex termed the polarisome is required for the assembly of a microfilament network, which is anchored at the leading edge of the budding daughter cell, and serves as a scaffold for the maternal acquisition of protein aggregates. One of the key polarisome components is actin, folded by the chaperonin CCT, which acts at high efficiency when deacetylated by *S. cerevisiae* SIR2 (ScSIR2) [53]. *S. cerevisiae sir2*-defective strains were defective in segregation of protein aggregates upon heat shock treatment compared to wild type cells. These findings suggest that one way in which SIR2 may control life span is through regulation of cytoskeletal components and distribution of invalid proteins further affecting a multitude of cellular functions.

Furthermore, *O*-acetyl-ADPR, which at least in yeast is almost exclusively produced by sirtuins [54] is a potential second messenger itself. The functions of *O*AADPR are currently obscure, however data from *in vitro* studies suggests that the molecule binds to several targets such as histone H2A.1.1 [55], cation channel TRPM2 [56] and SIR2 complexes themselves [57] possibly influencing their activity. Further studies are needed to elucidate the effects sirtuins exert through the unique *O*AADPR as well as the recently discovered *O*MaADPR and *O*SuADPR products.

The most well known and first studied site of action of many SIR2-like proteins is the end of the chromosome, the telomere where it acts in concert with other factors to negatively regulate gene expression (Fig. 4, bottom panel). The SIR2 complex in yeast consists of SIR2, SIR3 and SIR4 [58], with SIR3 and SIR4 being recruited first, by RAP1 and/or yKu70/yKu80 proteins. SIR2 is the last to bind and deacetylate lysines of histone H3 and H4, thereby creating a platform for further SIR3 and SIR4 binding and further expansion of heterochromatin regions [59,60]. Interestingly SIR3 recruitment by RAP1 has recently been shown to be sufficient for telomere clustering, a function of SIR3 independent of its deacetylase activity [61].

Multiple factors in yeast in addition to the SIR2 complex (and its recruitment factors) such as RIF1p, RIF2p [62], HDF1 and HDF2 [63], supplemented by processes such as histone modifications and histone variants, all combine to alter the access of transcription machinery to any given subtelomeric region in proportion to the proximity of the region to the telomere (reviewed in [64,65]). This so-called telomere position effect (TPE) or Position Effect Variegation (PEV) has been first described in *S. cerevisiae* as the reversible silencing of genes located near a telomere [66, for a review see 67], which is dependent on telomere length as well as the distance of the gene under study to the telomere [62,66,68,69].

Yeast SIR2 (ySIR2) is also known to play a crucial role at chromosome internal sites, notably in silencing at the silent mating-type loci HML and HMR [1,2,70], and tandem ribosomal RNA genes in yeast [71]. The association of ySIR2 with rDNA loci was observed to greatly decrease strictly local undesirable inter-rDNA recombination.

## 5 Roles of SIR2 proteins in protozoa

### 5.1 Two SIR2 proteins in *Plasmodium*

**Fig. 5:**
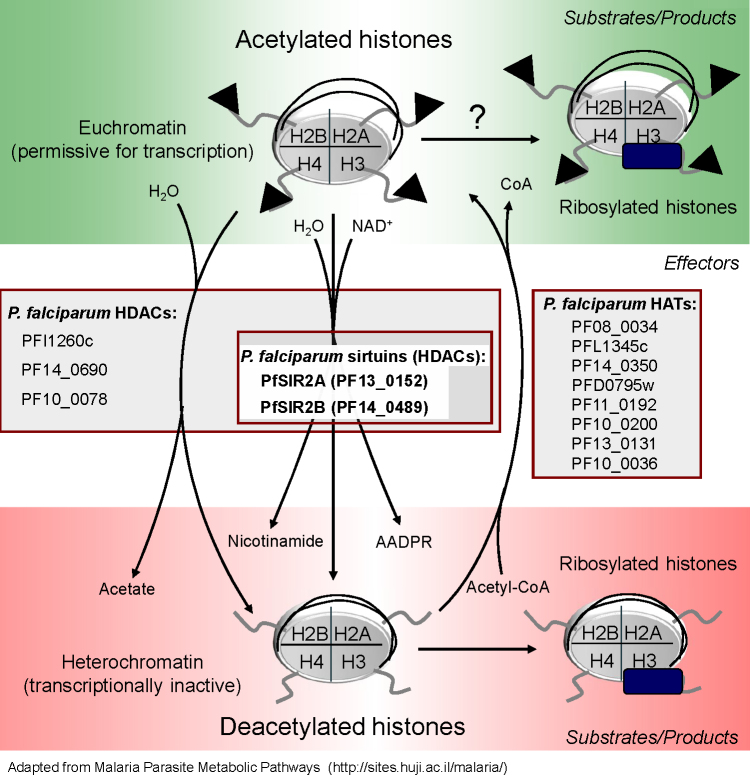
Acetylation/deacetylation pathway in *P. falciparum*. A vast number of proteins in a cell can undergo acetylation. Histones are one of the best studied targets of epigenetic modifications including acetylation. Acetylated (black triangles) histone tails (specifically Lys residues) are usually a part of transcriptionally-permissive (eu)chromatin (see top green panel). On these histones DNA is usually less “tightly” wrapped enhancing the accessibility of the transcription machinery. Histones can also be ribosylated. Collected data suggests that in *Plasmodium* ADP-ribosylation can be performed by sirtuins following deacetylation (see bottom red panel). However it has been recently shown for TbSIR2rp1 that ADP-ribosylation can occur independently of, though at a much slower rate than the deacetylation reaction. In addition to NAD^+^-dependent sirtuins (middle panel – *Effectors*) *P. falciparum* genome contains other histone deacetylases which are listed (HDACs). The product of deacetylation by any of the HDACs is a non-acetylated histone residue. Global deacetylation usually leads to chromatin condensation yielding transcriptionally inactive (hetero)chromatin. Additional deacetylation products are nicotinamide and 2′-*O*-acetyl-ADP ribose in case of sirtuins and acetate for the remaining HDACs. Histone acetylases (HATs) complete the cycle by performing histone acetylation using acetyl-CoA as a co-substrate. (For interpretation of the references to color in this figure legend, the reader is referred to the web version of the article.)

Several HDACs and histone acetylases (HATs) have been identified in *P. falciparum* genome (based on a recent review by Horrocks et al. [72] and PlasmoDB search; www.plasmodb.org) indicating the expected presence of functional acetylation/deacetylation processes similar to those found in other organisms (see Fig. 5, modified from Malaria Parasite Metabolic Pathways database). Of the five HDACs identified in the *Plasmodium* genome (PlasmoDB) two *sir2*-like genes were described based on sirtuin domain homology, *sir2a* and *sir2b*.

#### 5.1.1 Plasmodium SIR2A

*Plasmodium* SIR2A (class III) consist of little more than the PFAM SIR2 domain and SIR2A of different *Plasmodium* species share a protein sequence identity between 74 and 97% (CLUSTALW scores). *P. falciparum* PfSIR2A (or PfSIR2–13 encoded by PF13_0152/PF3D7_1328800; www.plasmodb.org) was identified through homology to the conserved core domain characteristic of the SIR2 proteins [73,74] and is the better characterised of the two SIR2 proteins in *P. falciparum*. Two research groups have independently shown that PfSIR2A is a mediator of transcriptional silencing at the subtelomeric regions [73,74]. This finding is important as *Plasmodium* genomes and telomere regions in particular contain a number of gene families encoding potential variant antigens families utilised by parasites to evade the host immune system in a process called antigenic variation. The multigene families include the *var* family (which appears to be unique to *P. falciparum*), *rifin*, some conserved families *e.g.* 235 kDa rhoptry protein, HAD-Hydrolase, pst-a, *etramp* [75], rodent parasite-specific families (*e.g. yir*/*cir*/*bir*, *pyst-a–pyst-d*, *pcst-f–pcst-h*). However and to date, only one of these gene families, the *var* family has been shown to be tightly regulated by sirtuin activity. In *P. falciparum* parasites the expression of *var* genes is mutually exclusive, [76,77], and switching from one *var* form is controlled epigenetically and does not involve DNA rearrangements [76,78]. Activation of a *var* gene imposes establishment of the permissive chromatin state, possibly involving physical translocation of the *var* gene within the nuclear periphery [74,79–81] One classification of *var* is based upon homologies in their promoter regions which are classified as upsA, upsB, upsC and upsE [82,83]. Transcriptome analysis of mutant parasites lacking expression of SIR2A, *ΔPfsir2a*, showed up-regulation of the steady state RNA level of many *var* genes compared to the wild type parasites [74] and the most significantly affected *var* genes were those controlled by *upsA* and *upsE* (which are all subtelomerically located and transcribed towards the telomere) and *upsC* promoters (which are chromosome-internal). A number of *rifin* multigene family members, generally located in close proximity to the affected *upsA*- and *upsE*-type *var* genes, were also found to be similarly affected in the absence of SIR2A expression. Subsequent analysis of independent clones of *P. falciparum* strain 3D7 lacking expression of SIR2A confirmed a broader spectrum of increased steady state RNA levels (∼2–3 fold up-regulation) of 8–10 *var* genes including *upsBA*- *upsBC* sub-type, with at least one protein product, VAR2CSA (*upsE*), detectable on the iRBC surface which normally requires selection for iRBC adhesion to chondroitin sulphate A to be detectable in wt parasites) [84]. Similar *var* sets were expressed by the different parasites lacking SIR2A expression. However in all studies no specific DNA motifs (in the promoter regions) were found that were common to the up-regulated *vars* which may imply epigenetic regulation of the up-regulated *vars*. Furthermore, switching of *var* gene transcription was slightly affected in the *SIR2A*-deficient parasites when compared to SIR2A-expressing parasites. Although the overall surface expression of one PfEMP1 member, VAR2CSA was higher in one parasite clone lacking SIR2A compared to wild type parasites (but lower than for the CS2 control line expressing large amounts of VAR2CSA) RNA transcripts levels for VAR2CSA were similar in *ΔPfsir2a* and CS2, which might indicate surface “saturation” or additional post-transcriptional mechanisms of *var* transcript control as mentioned above. Interestingly *in vivo* data from a study on Gambian *P. falciparum* isolates shows surprising *Pfsir2a* effects on *var* gene transcription [85]. *Pfsir2a* transcript levels were high and directly correlated with the *upsB*-type *var* transcripts abundance in the samples from severe malaria patients. In the same study *in vitro* over-expression of *Pfsir2a* agreed with the *in vivo* results, showing up-regulation of *upsB vars*.

Disease severity and certain *var* type expression have been linked in a number of studies (for instance [86–90]). Among several mathematical models developed for *P. falciparum* blood infection (*e.g.* [91–93]) one model assumes that above a threshold parasite density *var* expression becomes less tightly controlled [93]. Merrick et al. propose a model in which the severity of disease depends on a degree of host stress response, leading among others to the observed elevated *Pfsir2a* and *Pfsir2b* transcripts (and assumes that protein levels are accordingly increased), which in turn contribute to the deregulation of *var* gene transcription [85].

A study on a *P. falciparum* histone variant H2A.Z (PfH2A.Z) showed that the histone variant is associated with both active and silenced genes throughout the genome but that the PfH2A.Z distribution is highly consistent when it comes to *var* genes [94]. PfH2A.Z is present only at transcriptional start sites at active *var* promoters in wild type ring stage parasites [94]. However, in parasites lacking either SIR2A or SIR2B, the PfH2A.Z levels at promoters of the known repertoire of up-regulated *vars* were significantly increased compared with wt [94]. However, the acetylation status of H2A.Z has yet to be linked to its distribution in wt and Δ*sir2* mutants.

SIR2A-mediated histone modifications spread more than 50 kb into subtelomeric regions of *P. falciparum* chromosomes [73], significantly further than the estimated 3 kb in yeast. Further studies on *P. falciparum* telomere components led to a postulated fold-back or a t-loop telomere model [95,96] akin to a previously proposed model in yeast [97,98]. The models propose that telomeres fold back onto the subtelomeric regions, an event potentially adding to chromosome end stability, mediated by protein–protein interactions. *P. falciparum* origin-of-recognition complex 1 (PfORC1) was shown to physically associate with telomeric sequences [95] and could facilitate PfSIR2A telomeric and subtelomeric association. Heterochromatin protein 1 (HP1) is another element proposed to contribute to the t-loop formation as it has been shown to recognise H3K9me2 and H3K9me3 marks and bind to *P. falciparum* subtelomeric regions further facilitating heterochromatin formation [99]. ORC1 and HP1 were shown to interact with each other in Drosophila and Xenopus, and in Drosophila ORC1 is necessary for chromatin binding of HP1 [100–102]. The dynamic interaction of various components at *P. falciparum* chromosome ends is yet to be deciphered. Interestingly, telomere restriction fragment (TRF) analysis showed that PfSIR2A deletion causes an increase in average telomere from approximately 1.5 to 3 kb [12] although this had no obvious effect on parasite growth over 145 generations [13]. Conversely PfSIR2A over-expression in *in vitro*-cultured *P. falciparum* resulted in shortened telomeres (median length 1.45 kbp) at 34 days post-transfection compared to wild type (median length 1.72 kbp) [85]. It is worth noting that telomere length appears highly variable between different lab and field isolates (*e.g.* in this study [85]). Immuno-localisation studies show that the PfSIR2A appears as clusters localised both to the nucleolus (where it co-localises with PfORC1; [96]) and the nuclear periphery especially in early blood stages, and that the PfSIR2A foci co-localise with the telomeric clusters at the nuclear periphery in ring-stage parasites [73,96], as was previously observed in *S. cerevisiae* [103]. This distribution results from PfSIR2A binding to telomeres and subtelomeric regions, including at least four telomere-associated repeat elements (TAREs 1, 2, 3 and 6) implicated in Plasmodium chromosome clustering [73,96]. Telomere clustering in general has been implicated in spatio-temporal regulation of gene expression, and in promoting both homologous and ectopic recombination. Frequent double stranded breaks occur in subtelomeric regions of malaria parasite chromosomes [104], which at first glance might appear as disadvantageous to the parasite yet was later recognised as means of constant ectopic recombination-based diversification of the subtelomerically located antigenic var loci [78,105]. Ectopic recombination within the var members indicates formation of a temporary, recombination-permissive environment [74,80,81,106], a phenomenon that is plausibly largely enhanced in the absence of SIR2. Indeed, an inverse correlation between PfSIR2A presence and histone acetylation at var loci was observed, supporting the function of PfSIR2A as a major var-associated histone deacetylase. Upon activation of a specific telomere-associated var gene (var2csa), SIR2A is displaced from the promoter region initiating transition to transcriptionally active chromatin through histone hyperacetylation [73]. Interestingly PfSIR2A deletion and failure to deacetylate histones – particularly at the subtelomeric regions of the chromosomes – leads to a generally reduced incidence of the transcription suppressive H3K9me3 mark, including 5′ UTRs of vars and the rifin multigene family and might also contribute to a general increased transcriptional activity in these regions [107].

The function of SIR2A in *Plasmodium* was to date only studied in blood stages of *P. falciparum in vitro*, mainly through analysis of mutants lacking expression of SIR2A. Recently, similar *P. berghei* mutants have been generated lacking SIR2A. Preliminary analyses (unpublished data, Religa et al.) showed that the lack of expression of SIR2A causes an unexpected and complete block in parasite development at the ookinete-to-oocyst transition in the mosquito. The molecular basis of this phenotype is currently under analysis as is the effect of the lack of SIR2A on expression of members of subtelomerically located multigene families.

Recombinant PfSIR2A possesses the canonical function of a nicotinamide-sensitive protein deacetylase, more specifically a NAD^+^-dependent protein deacetylase [13,108]. PfSIR2A is able to partially substitute for the *sir2* gene absence in *S. pombe* proving that the *P. falciparum* sirtuin exhibits a degree of functional and structural conservation [13]. PfSIR2A performs protein deacetylation *in vitro* at a slow rate with *k*_cat_ values at an order of 10^−4^ s^−1^ [108,109] compared with a typical sirtuin deacetylation rate of 0.1–0.01 s^−1^. PfSIR2A can specifically deacetylate histone H3 lysine 9 and 14, as well as histone H4 at lysine 16 [109]. Curiously, in this study PfSIR2A exhibited preference for a human p300 (an auto-acetylating acetyltransferase regulated by human SIRT1 and SIRT2 by deacetylation) over highly conserved histone peptide targets, indicating a possible involvement of other molecular and/or spatial factors contributing to PfSIR2A activity *in vivo*. The *P. falciparum* SIR2A protein was also found to possess significant mono-ADP-ribosyltransferase activity on a variety of acetylated substrates including histones, BSA and itself *in vitro* [13]. Uniquely PfSIR2A possesses an acetyllysine-independent NAD^+^ glycohydrolase that is nicotinamide-insensitive although all the identified PfSIR2A functions are suspected to be catalysed by the same active site within the enzyme [109]. PfSIR2A has been reported to undergo multimerisation *via* C-terminal interactions, though unlike its trimeric yeast counterpart the complex was shown to disassemble to SIR2 monomers upon NAD^+^ binding [108,110].

Sirtuins were found to interact with a multitude of diverse proteins (see Section 4), including Alba proteins, which are a small family of RNA/DNA binding proteins. Purified recombinant PfSIR2A is able to co-precipitate recombinant *P. falciparum* Alba3 (PfAlba3) [111]. The interaction was confirmed using whole cell extracts and the recombinant PfAlba3 protein as a bait. Furthermore a peptide derived from the N-terminus of PfAlba3 can be deacetylated by PfSIR2A *in vitro*, preferably at the conserved Lys 22 – the site of alba modification by SIR2 in other systems [111]. PfSIR2A partially co-localises with PfAlba3 and PfAlba4 into spatially distributed foci in the *P. falciparum* nucleus, implying a potential role of Alba proteins in transcriptional regulation of telomeric/subtelomeric genes [111,112].

Given the role of PfSIR2A in *var* gene transcriptional regulation it is not surprising to find that PfSIR2A localises predominantly to the parasite nucleus and more specifically mostly to the nucleolus at ring stages [73]. Interestingly PFSIR2A shuttles to the cytoplasm as well, which is thought be a result of known PfSIR2A sumoylation [113] and although PfSIR2A possesses 3 theoretical sumoylation sites the precise site of such modifications has not yet been determined. Sumoylation is known to occur at Lys 734 in hSIRT1, which enhances its deacetylase activity [114]. It is currently unknown if PfSIR2A undergoes the extensive (at least 13 generally conserved sites) phosphorylation undertaken by the cyclinB/CDK1 complex as does hSIRT1 [115] and what the effects of such modification or lack thereof might be. Whilst sumoylation of SIR2A in *P. berghei* has not yet been studied, similar patterns of SIR2A nucleo-cytoplasmic shuttling have been demonstrated in both *P. berghei* blood stage asexual parasites and ookinetes (unpublished data Religa et al.). SIRT1 was also shown to be S-nitrosylated by nitrosylated Glyceraldehyde-3-phosphate dehydrogenase (SNO-GAPDH), which is transported to the nucleus following binding to Siah1 [116,117]. The S-nitrosylation, a protein post-translational modification, which inhibits the deacetylase activity of SIRT1 has not been formally shown for *Plasmodium* SIR2s.

Recent data suggests a further role for SIR2A proteins in *Plasmodium* as metabolic sensors: NAD^+^ levels are elevated in *P. falciparum*-infected red blood cells (RBCs) compared to non-infected RBCs, which are able to produce nicotinamide solely from either nicotinic acid or exogenous nicotinamide [118,119]. The NAD^+^ increase is consistent with extracellular nicotinamide depletion at late trophozoite and schizont stages of *P. falciparum* that could result from increased SIR2 activity. Such activity will be coupled to the levels of Acetyl-CoA which is derived from extracellular glucose [120] and is therefore also a direct reflection of the nutritional status of the parasite and its environment. Not surprisingly clinical isolates of *P. falciparum* were found to exhibit distinct physiological states, including one analogous to a starvation mode of *S. cerevisiae* [121], and *Pfsir2a* transcript level was found to be increased in the “starvation” group of parasites [85]. Both parasite *sir2a* and *sir2b* were up-regulated and in direct correspondence to high fever (≥37.5 °C) and blood lactate (≥4 mmol/L) in patients with severe malaria [85].

Further studies linking nutritional status with sirtuin activity and chromatin modification will determine the extent to which *Plasmodium* sirtuins play a role in environment sensing.

#### 5.1.2 Plasmodium SIR2B

*Plasmodium* SIR2B (PF14_0489) is a distant homologue of SIR2A and with a predicted size of 1304 aa almost five times larger than PfSIR2A [12]. SIR2B is present and syntenic in all sequenced *Plasmodium* spp. (PlasmoDB). All predicted *Plasmodium* SIR2Bs are large (>1000 aa) with a SIR2 superfamily domain (with a size of 384–512 aa) at the N-terminus of the protein. Based on this domain, the *Plasmodium* SIR2B are plausibly phylogenetically placed in the class IV sirtuins [12 and see Fig. 1] and the domain most closely resembles the SIRT7 sub-type conserved domain which is found in human sirtuin SIRT6, SIRT7 and several bacterial homologues. The mammalian SIRT6 protein was first shown to exhibit predominantly ADP-ribosyltransferase activity [28], and SIRT6^−/−^ mice show a premature ageing phenotype caused by a defect in the Base Excision Repair pathway [122]. SIRT6 does possess deacetylase activity highly specific to histone H3 lysine 9 (H3K9Ac) and lysine 56 of histone H3 (H3K56Ac) – histone modifications through which SIRT6 is thought to affect genomic stability [123–126]. hSIRT6 participates in the DNA double-strand break (DSB) repair process through a recently found interaction with C-terminal binding protein interacting protein (CtIP) [123]. Importantly it is the SIRT6-dependent deacetylation of CtIP which regulates the DNA end resection. Interestingly, SIRT7 is the only mammalian sirtuin that preferentially localises in nucleoli [127] and it has been shown to be an activator of RNA polymerase I transcription of rRNA genes (rDNA) by interactions with RNA polymerase I and histones [128]. The sub-cellular location of PfSIR2B has not yet been reported. Despite being paralogous, *Plasmodium* SIR2A and SIR2B are highly divergent, indicating distinct functions. The NAD^+^-binding sirtuin domain at the N terminus of both proteins is the only stretch of high amino acid homology, as presented in Fig. 2B. The activities of SIR2B might be anticipated to be predominantly nuclear by analogy with the most extensively studied members of this clade (namely SIRT6 and SIRT7).

Interestingly, mutant parasites that lack expression of PfSIR2B implicate it in transcriptional silencing of a complementary subset of *var* genes to SIR2A, in this instance acting predominantly at subtelomeric *vars* of the *upsB* type [12]. However unlike in the *pfsir2a*^−^ parasites, the de-regulated *var* promoters in the *pfsir2b*^*−*^ mutant were significantly enriched in the PfH2A.Z histone variant only at early blood stages (rings) and not in schizonts (late blood stage) [94] (also see the *Plasmodium* SIR2A section). In contrast to PfSIR2A, the absence of PfSIR2B expression does not affect telomere length, as measured by telomere restriction fragment (TRF) analysis, which might indicate that this protein is present at telomeres at relatively low concentrations and/or that it might exert other yet unidentified effects in the parasite. No biochemical analysis of the sirtuin domain of *Plasmodium* SIR2B has yet been reported leaving open the possibility that SIR2B may exhibit different NAD^+^-dependent deacetylation/ADP-ribosylation rates than SIR2A, as well as additional novel functions yet to be discovered.

### 5.2 Other apicomplexans

Do SIR2-like proteins of other apicomplexan and indeed protozoan parasites play the same role as those of *Plasmodium* and control the clonal expression of surface antigens that are exposed to selection (*e.g.* the host immune system)? Unfortunately, to date there is no experimental support for this hypothesis although the possibility has yet to be thoroughly addressed in most parasites. Indeed little or no functional data exists for other apicomplexan proteins regarding the role(s) of sirtuins. A brief outline of the different modes of antigenic variation in the most well-studied protozoan parasites follows with indications of how SIR2 might be expected to play a role.

The apicomplexan parasite *Babesia bovis* infecting cattle usually prevails in the host for many years at least in part through shuffling of the heterodimeric variant erythrocyte surface antigen-1 (VESA-1) on the surface of infected erythrocytes [129,130]. Unlike in *Plasmodium* and more akin to African Trypanosomes (see below), the switching process of *B. bovis ves1* surface antigens involves DNA rearrangements, in a process called segmental gene conversion [131] (see Suppl. Fig. 1B). Nevertheless, it has been proposed that both DNA rearrangements and epigenetic mechanisms play a role in transcriptional control and switching of expression of *ves1* genes (*var*-gene switching in *P. falciparum* does not involve DNA rearrangements). The *B. bovis* genome appears to contain a single sirtuin gene resembling SIR2Bs of other Apicomplexa (see Table 1 and Fig. 2B). Currently no data is available on the localisation of BbSIR2 and its possible function in epigenetic control of transcription of variant antigens.

The *Toxoplasma gondii* and *Neospora caninum* are most similar to *Plasmodium* with respect to the presence of sirtuins in the genome in that they contain genes that encode both SIR2A and SIR2B-like sirtuins (see Table 1). *Theileria* spp. and *Cryptosporidium* spp. both encode single SIR2A-like proteins. The *C. hominis* single SIR2 protein was shown to localise in the nucleus when expressed ectopically in *D. discoideum* [132]. No studies were performed on endogenous SIR2 localisation in *C. hominis*. The *Eimeria tenella* may encode two SIR2-like proteins though current genome assembly state only allows for confident assignment of one of them to SIR2A type (ETH_00033350) whilst the other based on the small portion of the available sequence resembles a SIR2B type (ETH_00041870; see Fig. 2B).

Although antigenic variation has not been shown in the remaining Apicomplexa mentioned in this review, in most parasites there is evidence for multigene families located both subtelomerically and chromosome-internally that might feasibly be influenced by sirtuin activity. Surface immunogenic molecules such as glycosylphosphatidylinositol (GPI)-anchored surface antigens (SAGs) belonging to the SAG1-related sequences (SRS) superfamily capable of inducing host immune responses are present at least in *Toxoplasma*, *Neospora* and *Eimeria* [133–136], with members expressed both across stages and with stage-specificity [137–143]. *Theileria* spp. genomes contain several *Theileria*-specific gene families mostly of unknown function, including sub-telomerically located *SVSP* and *Sfil* genes, as well as chromosome-internal *TashAT* and *Tar*/*Tpr* families [144, and references therein]. In *Cryptosporidium* several surface mucin-like glycoproteins were identified mediating host invasion and inducing antibody responses (*e.g.* [145–147]).

The molecular mechanisms underlying the host immune evasion for the majority of Apicomplexan parasites therefore, remain to be entirely understood. Multigene families, of which some could be expressed in a clonally variant manner, do seem to give raise to the diverse surface antigens at the core of host-parasite interactions. The chromosomal location of the families is mostly unassigned due to incomplete genome assemblies and characterisation. However the plausible theory is that when/if these families are located subtelomerically, they can undergo beneficial diversification, as well as efficient and swift switching of members at a transcriptional level. Any role sirtuins may play in the regulation of these diverse gene families in the various members of the apicomplexans awaits first the clarification of the organisation of these families and generation of the appropriate tools for their analysis.

### 5.3 Other protozoan parasites

#### 5.3.1 Trypanosomatids

##### 5.3.1.1 Trypanosoma brucei

*T. brucei*, the causative agent of African sleeping sickness, varies VSG antigen expression both by reorganisation in the *vsg* family DNA sequence *via* duplication transposition at the telomerically located expression linked sites (ELS) [148] and transcriptional *in situ* switching between different ELS of which there are about 20 (Suppl. Fig. 1C). Only one *vsg* promoter is active at any given time driving transcription of a single *vsg* gene. The diversity of v*sg* is driven *in situ* by extensive recombination between the 1000's of *vsg* (pseudo)genes which form the repertoire reservoir, a process that will involve extensive DNA repair [149].

*Trypanosoma brucei* has three sirtuins genes, encoding TbSIR2-related proteins (TbSIR2rp1–3, see Table 1) and only TbSIR2rp1 has been shown to localise to the nucleus, where it can participate in DNA repair as well as RNA polymerase I-mediated transcription repression of subtelomeric genes both in insect and blood stages [150,151]. TbSIR2rp1 belongs to class I sirtuins, together with hSIRT1–3. RNAi-mediated knockdown of TbSIR2rp1 did not affect blood stage VSG gene expression and the tubulin acetylation state remained unchanged (hSIRT2 is known to target alpha-tubulin). Furthermore *T. brucei* SIR2rp1 was shown to associate with subtelomeric sequences [150], silencing genes only within 2 kb from a telomere that did not possess an ELS more akin to *S. cerevisiae* than *P. falciparum* where SIR2A is found at least 50 kb from a telomere. Although, ELS-linked *vsg* genes are regulated independently with a cluster of other subtelomeric genes, SIR2rp1-deficient *T. brucei* parasites exhibit a general de-repression of Pol I-transcribed genes but *vsg* genes remain silent indicating other repression mechanisms are operative at *vsg* loci [150]. It is therefore probable that mutually exclusive expression of VSG antigens is dependent on other non-sirtuin-based mechanisms such as base J [152,153] although chromatin remodelling factors other than sirtuins may also play a role. Biochemically, recombinant TbSIR2rp1 is able to transfer ADP-ribose to histones, BSA as well as itself [150] and exhibits deacetylation activity 5 fold higher than ADP-ribosyltransferase activity [25]. The additional two SIR2-like proteins of *T*. *brucei* both localise to the mitochondrion and share approximately 45% nucleotide homology with *Tbsir2rp1*. Disruption of either TbSIR2rp2 or TbSIR2rp3 individually showed no detectable differences between wild type and mutant parasites in growth rate and differentiation *in vitro* to the insect stage [151].

##### 5.3.1.2 *Trypanosoma cruzi* and *Leishmania* spp.

Antigenic variation in *Trypanosoma cruzi* and *Leishmania* spp., which infect erythrocytes and macrophages respectively in their vertebrate hosts, is not well understood. The parasites present several types of proteins on their surface. For instance lipophosphoglycans (LPGs) and GPI-anchored proteins, found on the surface of *Leishmania* parasites and Mucin-like proteins presented on *T. cruzi*-infected cells are the basis for antigenicity and regulate the parasites’ survival and virulence. However, these proteins are single copy and not dispersed to the telomeres, therefore there is no expectation that SIR2 might influence their expression by the mechanisms outlined for some other protozoan parasites. Antigenic variation in these parasites stems rather from the somewhat stochastic nature of carbohydrate addition to these protein templates resulting in a non-homogenous surface possibly of spectral antigenicity as the interface with the immune system.

Freed from this role in antigenic variation, SIR2 proteins in these species offer systems of study for additional activities and roles of SIR2 sirtuins in parasitic protozoa.

*T. cruzi* possesses 2 sirtuin-like genes TcSIR2rp1 (class I), and TcSIR2rp3 (class III), missing a TbSIR2rp2-like gene (Table 1 and [154]). The functions and localisations of these sirtuins are currently unknown.

*Leishmania* spp. contain three predicted sirtuins in their genomes (see Table 1 and [155]). Recombinant *L. infantum* SIR2rp1 (LiSIR2rp1) was the first sirtuin definitively shown to ADP-ribosylate α-tubulin causing either tubulin depolymerisation or inhibiting tubulin assembly as well as deacetylating α-tubulin [156]. *L. major* LmSIR2rp1 physically interacts with a heat shock protein HSP83 (a mammalian orthologue of HSP90), although it does not seem to deacetylate the HSP83 itself [157]. *L. amazonensis* SIR2rp1 (LaSIR2rp1) exhibits some substrate specificity in that it was able to deacetylate a major acetylated protein (∼50 kDa) in promastigote extracts but not acetylated BSA [158]. LiSIR2rp1 was found principally in the cytoplasm and partially co-localised with the microtubule network, but is reported to be found in cytoplasmic granules and in excreted-secreted antigens (ESA), as is LmSIR2rp1 and LaSIR2rp1 in both promastigotes and amastigotes [158,159]. LiSIR2rp1 like TbSIR2rp1 and PfSIR2A also auto ADP-ribosylates itself in the presence of histones and exhibits both NAD^+^-dependent deacetylase activity as well as ADP-ribosyltransferase activity [159,160]. LaSIR2rp1 is a sirtuin-type deacetylase though the ADP-ribosyltransferase activity was not formally shown [158]. LaSIR2rp1 was proposed to be glycosylated *in vivo* since it was immuno-detected in lectin affinity purified promastigote and amastigote extracts [158]. Over-expression of LmSIR2rp1 in different *Leishmania* species significantly delayed apoptosis-like death at different parasite stages [160]. *Lisir2rp1*^+/−^ amastigotes but not promastigotes exhibit defects in multiplication both *in vitro* and *in vivo* in Balb/c mice. LiSIR2rp1 appears to be essential as *Lisir2rp1*^−/−^ mutants could be obtained only with simultaneous introduction of SIR2rp1 expressing episomes [161]. Uniquely amongst the SIR2 proteins of protozoan parasites, LiSIR2rp1 has been shown to influence the host immune system through being a component of ESA: it specifically induces proliferation of activated B cells leading to increased surface expression of CD40 and CD86 which in turn may induce T cell activation thereby initiating an adaptive immune response [162,163]. Indeed it appears that LiSIR2rp1 stimulates many arms of the immune response as it can induce maturation of dendritic cells (DCs) and activate macrophages. LiSIR2rp1 appears to mediate its effects on B cells and DCs through Toll-like receptor 2 (TLR2) both *in vitro* and *in vivo* although macrophage activation is TLR2-independent as shown in TLR2-deficient mice. It is noteworthy that *Leishmania* surface LPGs stimulate immune signalling pathways through binding to TLR2 thereby activating macrophages and natural killer (NK) cells [164,165]. In the absence of *Lisir2rp1*^−/−^ parasites it is not possible to investigate the importance of LiSIR2rp1 to eliciting a protective immune response in the host other than through tolerisation.

##### 5.3.1.3 Giardia lamblia

*G. lamblia* undertakes clonal antigenic variation [166] as means of evading the host immune system. This intestine resident parasite species contains approximately 190 homologous, variant-specific surface protein (*vsp*) genes dispersed throughout the genome. Furthermore, multiple copies of a particular *vsp* gene might be tandemly arranged on a chromosome region [167], as well as duplication of upstream and downstream regions from one *vsp* to another [168]. Importantly and in contrast to the general trend in protozoa described above, the *vsp* genes are predominantly non-subtelomeric [169]. Switching of expression of *vsp* is not associated with DNA rearrangements suggesting the participation of epigenetic mechanisms such as histone acetylation in the regulation of *vsp* transcription [170–172] (Suppl. Fig. 1D). However, it appears that the major mechanism to effect antigenic variation does not involve control of transcription but rather RNA interference. A cytosolic ribosome-associated RNA-dependent RNA polymerase (RdRP) together with Dicer and Argonaute degrade all *vsp* RNA transcripts except from the predominant one which is translated and expressed on the parasite surface [173]. A targeted knock down of Dicer and RdRP resulted in the loss of mutually exclusive expression. How the predominant *vsp* transcript bypasses the silencing machinery and how switching from one *vsp* to another occurs in the first place remain to be described.

*Giardia* is one of the most ancient eukaryotes, which is consistent with the observation that two of *Giardia* five sirtuins (GL50803_16569 and GL50803_6942, see Table 1) belong to class U and are quite distant from other sirtuins (Fig. 1). Our analyses relate both sirtuins more closely to class U of mostly bacterial sirtuins. At present there is no experimental characterisation of the role of sirtuins in *Giardia* spp.

Thus to date, the role of SIR2 in protozoal antigenic variation is only unambiguously established for *P. falciparum*. Research is needed on other *Plasmodium* species as well as other apicomplexan parasites such as *Toxoplasma*, *Neospora*, *Eimeria*, *etc.* which all share very similar sirtuins to *Plasmodium* SIR2 to determine if sirtuins play a similar role in the epigenetic control of expression of subtelomeric genes in other Apicomplexa or if is limited to *Plasmodium* species and perhaps just *P. falciparum*. Unpublished research has revealed additional roles and an overt cytoplasmic distribution of SIR2A in *P. berghei* ookinetes emphasising the multifunctional nature of this protein class (unpublished data, Religa et al.). Therefore, in addition to the already identified SIR2 functions in Apicomplexa and other protozoans surely many more, not necessarily related to antigenic variation are likely to be uncovered in all protozoan parasites.

## 6 Are sirtuin inhibitors/activators potential anti-parasitic drugs?

### 6.1 Inhibitors

General sirtuin inhibitors are already available and under development (for a review see [174]) and their effects on parasite survival might be addressed experimentally. The use of SIR2 inhibitors against *P. falciparum* is likely to be restricted to therapeutic strategies that seek to enhance the immunity of the host to the parasite and are unlikely to be specific mediators of direct parasite killing. However should PfSIR2A prove to be essential for parasite transmission as it is in the rodent parasite *P. berghei* (Religa et al., unpublished data) then an added dimension of such therapy might be the reduced transmission of parasites from treated individuals.

The most common sirtuin inhibitor nicotinamide has a moderate effect on PfSIR2A *in vitro* acting in the μM range albeit in a dose-dependent manner. Complete inhibition of SIR2 activity was reached only at a level of 10 mM nicotinamide and therefore pharmacologically unusable [13]. Nicotinamide showed anti-malarial activity against cultured *P. falciparum* blood stages [175] at high concentrations (IC_50_ value of 10 mM) but this effect is more likely to be due to off-target effects in view of the non-essential nature of SIR2 in *Plasmodium* [12,74]. Similarly a novel competitive inhibitor surfactin with a relatively low IC_50_ value against recombinant PfSIR2A *in vitro* (∼35 μM) was able to inhibit *P. falciparum* blood stage growth at a low concentration (10 μM) [108]. However the surfactin inhibitory effect may also be exerted through SIR2-independent effects on the parasite since it was shown to induce apoptosis and cycle arrest-mediated killing of human colon carcinoma cells [176]. Sirtinol, a proven sirtuin-specific inhibitor was found to inhibit growth of *L. infantum* axenic amastigotes (IC_50_ value ∼60 μM as measured by DNA fragmentation), but not promastigotes *in vitro*, independently of a concentration of the inhibitor used [177]. Interestingly episomally-expressed LmSIR2 partially alleviated the effects of sirtinol. A highly sirtuin-specific inhibitor bisnaphthalimidopropyl (BNIP) has been recently developed against the class I *Leishmania* LiSIR2RP1 [178]. The inhibitory action of BNIP is in the low micromolar range (5.7 μM) against the purified LiSIR2RP1 and the compound is selective towards the parasite enzyme when compared with human SIRT1.

### 6.2 Activators

The use of activators in the studies of parasitic protozoa can be potentially beneficial, *e.g.* in studies of antigenic variation mechanisms and effects (in case of *P. falciparum*) or biological consequences of extended parasite life span (*e.g. Leishmania*). A number of sirtuins activators have been developed in the recent years following the identification of the possible link of SIR2 to life-span regulation in a number of organisms (see for instance [179]). The most renowned sirtuin activating compound (STAC) is a phytochemical polyphenol resveratrol which prolongs life-span of nematode worms, fruit flies, rodents (for instance [180–183]) at least in part through SIR2-like protein activation. The effects of such activators on parasitic protozoa have yet to be elucidated. Akin to activation, over-expression of sirtuins appears to be advantageous in most organisms studied to date including yeast, worms, flies, mammalian cells [184–188]. There is limited knowledge of the effects of SIR2 over-expression in protozoa. Vergnes et al. [160] showed that *L. infantum* parasites over-expressing *L. major* (LmSIR2) or *L. infantum* SIR2 (LiSIR2) exhibited increased survival of stationary phase amastigotes (vertebrate host). Promastigotes (insect stages) of *T. brucei* with 2–3 fold increased SIR2 (TbSIR2RP1) expression levels augmented parasite resistance to DNA damage [150].

In general, it seems that protozoa also flourish in an excess of sirtuins and perhaps points to some commonality of function aside from species or protozoa-specific roles of this intriguing family of proteins.

For further general reading on sirtuin modulators please refer to a review by Mahajan et al. [189].

## 7 Future perspectives

It has become clear in recent years that protein acetylation serves a central regulatory role on protein function and within this sphere of activity the role(s) of sirtuins is (are) likely to prove critical. However, much remains to be learned about the roles and functions of sirtuins in apicomplexans. Uncovering critical parasite-specific sirtuin functions could be crucial to understanding parasite nutritional and survival strategies in response to changing environments (*e.g.* host nutritional status) within specific hosts, and would help determine if this class of proteins are viable drug targets. In addition both apicomplexans and *Trypanosomatidae* which are relatively easily maintained and manipulated in a laboratory, might serve as models to extend our current knowledge of conserved features of sirtuin biology. A variety of effects exerted by sirtuin inhibitors and activators (or sirtuin deletion or over-expression) on a number of organisms indicates that sirtuins are involved in a wide range of cellular functions and have important roles in all kingdoms of life. Deciphering how these fascinating proteins coordinate and influence multiple pathways exerting micro- (intracellular) and macro- (intercellular, whole organism) effects is one of our next challenges. This knowledge is necessary in order to properly utilise sirtuin inhibitors and/or activators, which might serve as potentiators of other anti-parasitic drugs targeting related processes where not potent enough on their own.

## Figures and Tables

**Figure:**
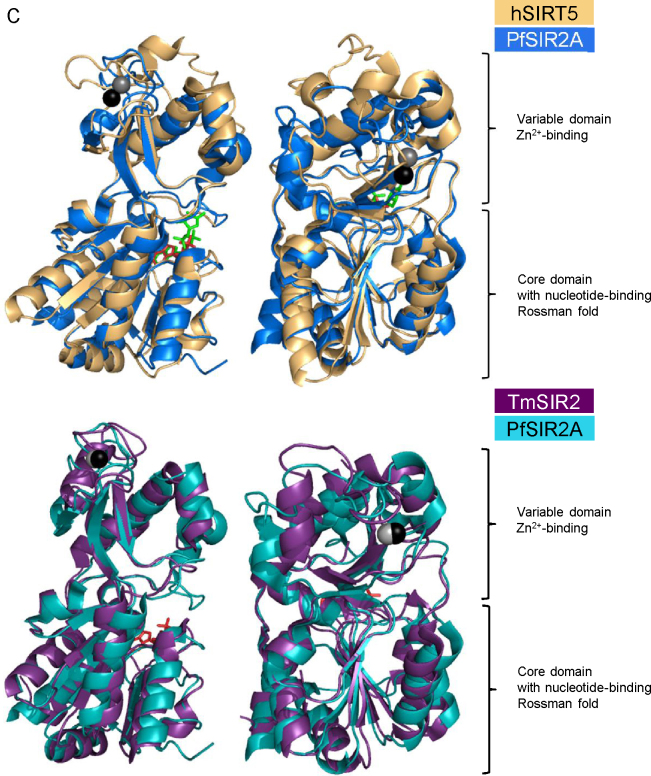


**Table 1:** Summary of sirtuins complement of parasitic protozoa genomes.

Organism	No. of sirtuins^a^	Gene ID^b^	Phylogenetic classes	Localisation^c^	Data source
Apicomplexa					
*Plasmodium* spp.	2	*P. falciparum*: PF13_0152 (SIR2A); PF14_0489 (SIR2B)	III (SIR2A), IV (SIR2B)	Nucleus (A, B?), cytoplasm (A, B?)	PlasmoDB (v8.2)
*Toxoplasma* spp.	2	*T. gondii VEG*: TGVEG_068040 (SIR2A); TGVEG_108780 (SIR2B)	III (SIR2A), IV (SIR2B)	n.d.	ToxoDB (v7.2)
*Neospora caninum*	2	NCLIV_045990 (SIR2A); NCLIV_038820 (SIR2B)	III (SIR2A), IV (SIR2B)	n.d.	ToxoDB (v7.2)
*Theileria* spp.	1	*T. annulata*: TA20415 (SIR2B)	IV (SIR2B)	n.d.	PiroplasmaDB (v1.1)
*Cryptosporidium* spp.	1	*C. hominis*: Chro.70234 (SIR2A?)	U? (SIR2A?)	Nucleus^d^	CryptoDB (v4.6)
*Babesia bovis*	1?	BBOV_I003070 (SIR2B)	IV (SIR2B)	n.d.	PiroplasmaDB (v1.1)
*Eimeria tenella*	1–2?	ETH_00033350 (SIR2A); ETH_00041870 (SIR2B?)	III (SIR2A), IV (SIR2B)	n.d.	ToxoDB (v7.2)

Sarcomastigophora					
*Trypanosoma brucei*	3	Tb927.7.1690 (TbSIR2rp1); Tb927.8.3140 (TbSIR2rp2); Tb927.4.2520 (TbSIR2rp3)	I (TbSIR2rp1), II (TbSIR2rp2), III (TbSIR2rp3)	Nucleus (TbSIR2rp1), mitochondrion (TbSIR2rp2, TbSIR2rp3)	TriTrypDB (v4.0)
*Trypanosoma cruzi*^e^	2	Tc00.1047053507519.60 (TcSIR2rp1); Tc00.1047053506559.80 (TcSIR2rp3)	I (TcSIR2rp1), III (TcSIR2rp3);	n.d.	TriTrypDB (v4.0)
*Leishmania* spp.	3	*L. infantum*: LinJ.26.0200 (LiSIR2rp1), LinJ.23.1450 (LiSIR2rp2); LinJ.34.1900 (LiSIR2rp3)	I (LiSIR2rp1), II (LiSIR2rp2), III (LiSIR2rp3)	Nucleus, cytoplasm (granules) (LiSIR2rp1)	TriTrypDB (v4.0)
*Giardia lamblia*	5	GL50803_10708 (GlSIR2_1), GL50803_10707 (GlSIR2_2); GL50803_16569 (GlSIR2_3); GL50803_11676 (GlSIR2_4); GL50803_6942 (GlSIR2_5)	I (GlSIR2_1, GlSIR2_2, GlSIR2_4?), U (GlSIR2_5, GlSIR2_3?)	n.d.	GiardiaDB (v2.5)
*Trichomonas vaginalis*	11	TVAG_549940; TVAG_409810; TVAG_026260; TVAG_319320; TVAG_480900; TVAG_190210; TVAG_362260; TVAG_413390; TVAG_146810; TVAG_016210; TVAG_146820; TVAG_256040	U (TVAG_016210), I (remaining sirtuins)	n.d.	TrichDB (v1.3)

n.d. = not determined.

a Based on the available sequence data as of April 2012.

b IDs of a representative species are shown.

c Please note that localisation information is not complete for all sirtuins in most species.

d Localisation determined in *Dictyostelium discoideum* using a plasmid carrying *C. hominis sir2* gene.

e Non-Esmeraldo-like.
