# Supplementary material for: Sirtuins of parasitic protozoa: In search of function(s)
Source: Mol Biochem Parasitol. 2012 Oct;185(2-2):71–88. doi: 10.1016/j.molbiopara.2012.08.003 (PMC3484402; doi:10.1016/j.molbiopara.2012.08.003)
Supplement: Supplementary file 1 [file mmc1.pptx]

## Slide 1
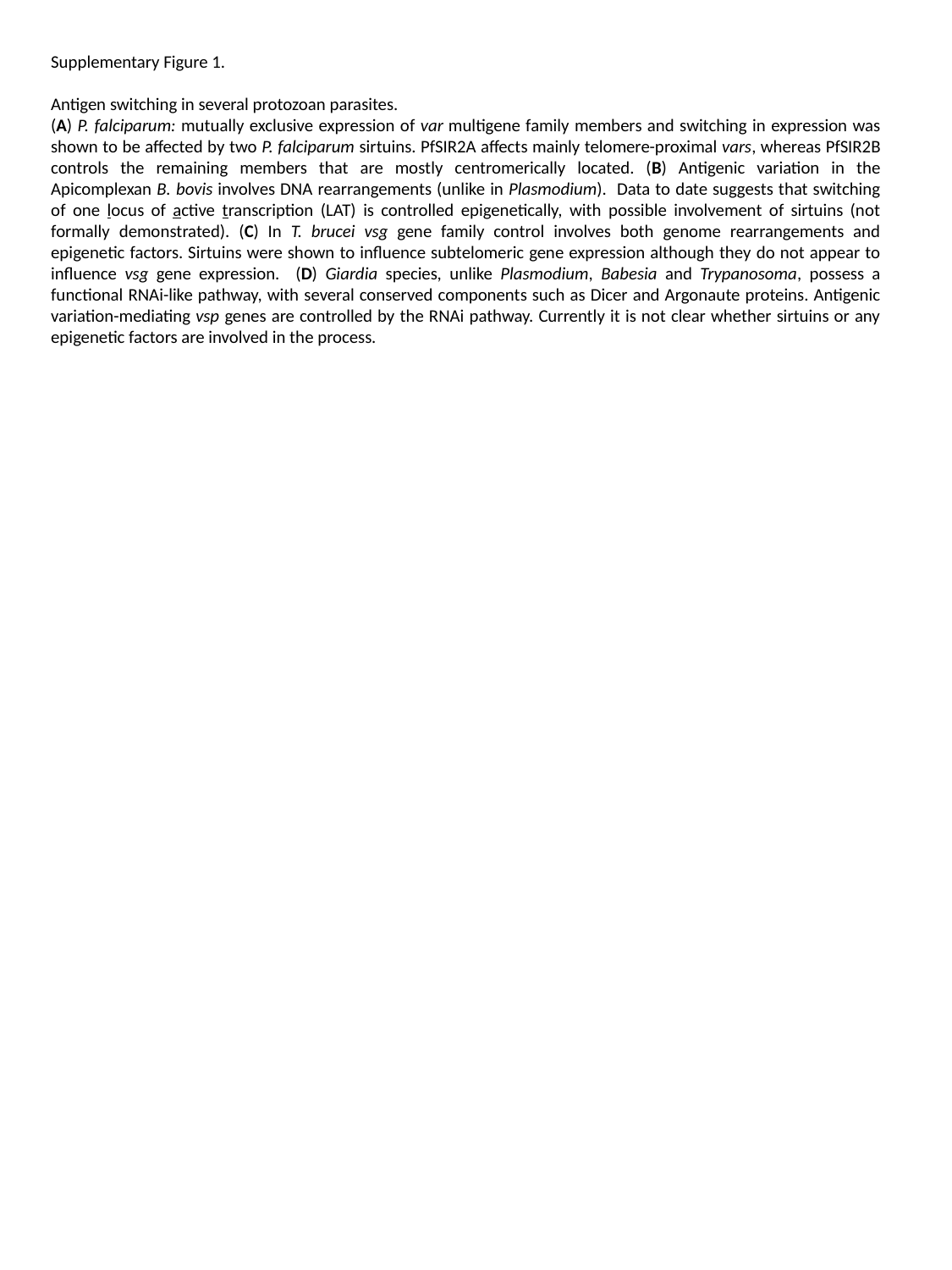

Supplementary Figure 1.
Antigen switching in several protozoan parasites.
(A) P. falciparum: mutually exclusive expression of var multigene family members and switching in expression was shown to be affected by two P. falciparum sirtuins. PfSIR2A affects mainly telomere-proximal vars, whereas PfSIR2B controls the remaining members that are mostly centromerically located. (B) Antigenic variation in the Apicomplexan B. bovis involves DNA rearrangements (unlike in Plasmodium). Data to date suggests that switching of one locus of active transcription (LAT) is controlled epigenetically, with possible involvement of sirtuins (not formally demonstrated). (C) In T. brucei vsg gene family control involves both genome rearrangements and epigenetic factors. Sirtuins were shown to influence subtelomeric gene expression although they do not appear to influence vsg gene expression. (D) Giardia species, unlike Plasmodium, Babesia and Trypanosoma, possess a functional RNAi-like pathway, with several conserved components such as Dicer and Argonaute proteins. Antigenic variation-mediating vsp genes are controlled by the RNAi pathway. Currently it is not clear whether sirtuins or any epigenetic factors are involved in the process.

## Slide 2
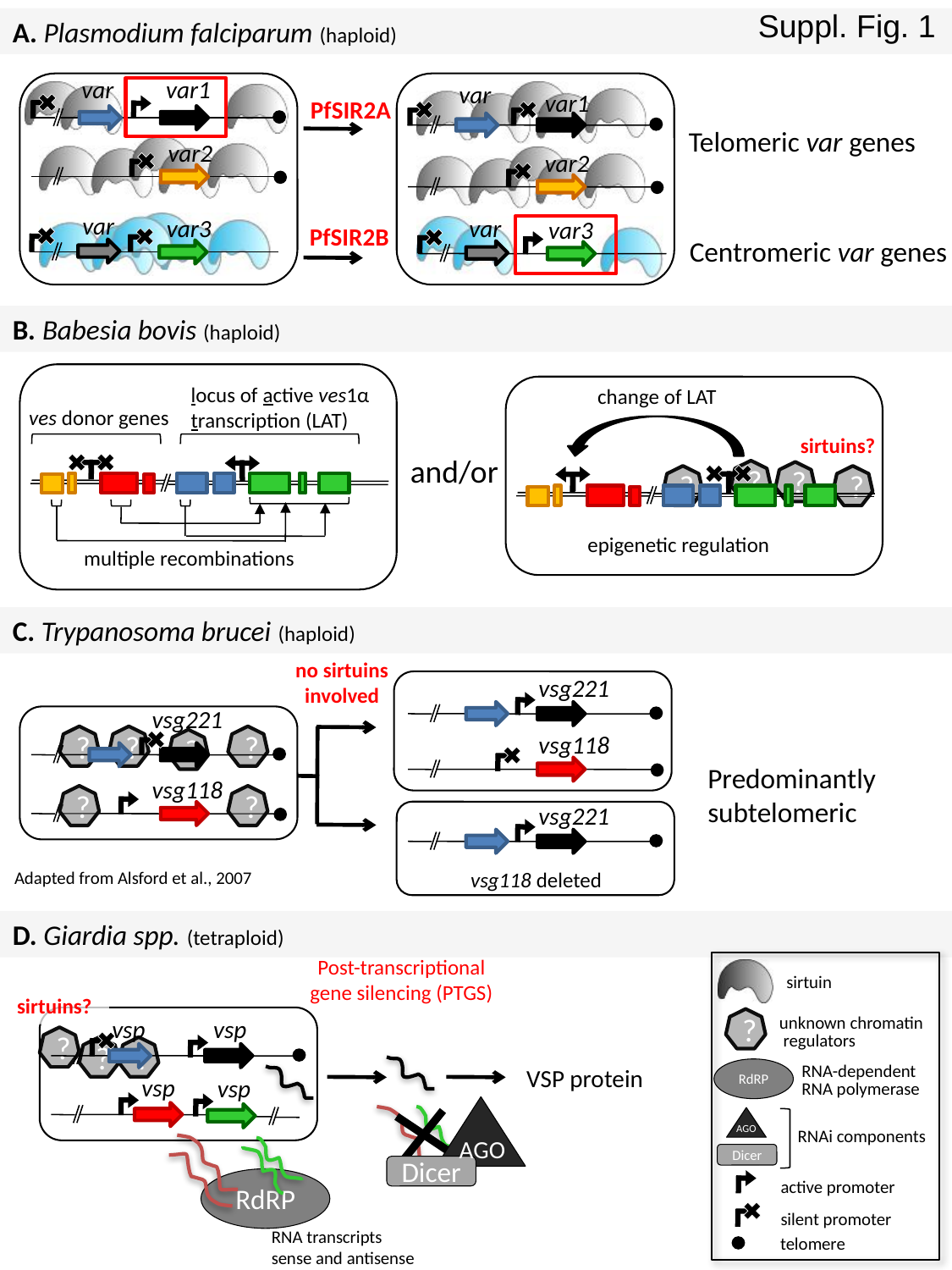

Suppl. Fig. 1
A. Plasmodium falciparum (haploid)
var1
var
var
var1
PfSIR2A
Telomeric var genes
var2
var2
var
var3
var
var3
PfSIR2B
Centromeric var genes
var1
B. Babesia bovis (haploid)
locus of active ves1α transcription (LAT)
change of LAT
ves donor genes
sirtuins?
and/or
?
?
?
?
var1
var1
epigenetic regulation
multiple recombinations
C. Trypanosoma brucei (haploid)
no sirtuins involved
vsg221
vsg221
vsg118
?
?
?
?
Predominantly
subtelomeric
vsg118
?
?
vsg221
vsg118 deleted
Adapted from Alsford et al., 2007
D. Giardia spp. (tetraploid)
Post-transcriptional gene silencing (PTGS)
sirtuin
sirtuins?
vsp
vsp
?
unknown chromatin
 regulators
?
?
?
VSP protein
RNA-dependent
RNA polymerase
RdRP
vsp
vsp
AGO
RNAi components
AGO
Dicer
Dicer
RdRP
active promoter
silent promoter
RNA transcripts
sense and antisense
telomere
